# Patient-Assessed Outcomes following Temporal Bone Fractures

**DOI:** 10.3390/diagnostics12020547

**Published:** 2022-02-21

**Authors:** Elias Antoniades, George Psillas, Konstantinos Polyzoidis, Ioannis Patsalas

**Affiliations:** 1 First Neurosurgical Clinic, AHEPA University Clinic, 546 36 Thessaloniki, Greece; eliasantoniad@yahoo.gr (E.A.); polyzoik@auth.gr (K.P.); patsalas@auth.gr (I.P.); 2 First Academic ENT Department, Aristotle University of Thessaloniki AHEPA Hospital, 546 36 Thessaloniki, Greece

**Keywords:** temporal bone fractures, quality of life, hearing loss, facial nerve, dizziness, electroneurography, excitability test

## Abstract

The long-term impact of neurotological symptoms after a temporal bone fracture (TBF), including facial nerve palsy (FP), hearing loss, tinnitus, and dizziness on the quality of life of patients is often underevaluated. Thus, we retrospectively assessed 30 patients with TBF (26 men and 4 women) in our university tertiary referral center. They participated from injury onset to the final follow-up, over an 18-month period. Quality of life was estimated using validated questionnaires, such as the Facial Disability Index (FDI: physical and social), Hearing Handicap Inventory (HHI), Tinnitus Handicap Inventory (THI), and Dizziness Handicap Inventory (DHI). The FDI score was significantly worse in patients with severe initial (for physical FDI) and final facial palsy (for both physical and social FDI), mainly with immediate onset. The HHI score was statistically worse in patients with mixed hearing loss compared to those with conductive or sensorineural hearing loss and in those with profound hearing loss vs. normal hearing. The mixed TBF and the severity of hearing loss (especially profound hearing loss) were correlated with HHI, THI and DHI score values. In the long-term period after a TBF, moderate or severe facial palsy, mainly with immediate onset, may cause psychological distress, more easily resulting in social disability than functional impairment. Mixed TBF and mixed or profound hearing loss may also negatively influence quality of life.

## 1. Introduction

Temporal bone fracture (TBF) represents 14–22% of cranial fractures [1,2,3]. The temporal bone is the thickest bone in the body, requiring excessive force to fracture; a TBF may occur in fresh human cadavers, when the applied force to the lateral skull is about 6000–8000 Newtons (equivalent to 600–800 Kilograms-force) [4], increasing the risk of neurovascular injury.

Conventionally, TBFs are divided into longitudinal, transverse, and mixed types, depending on the direction of the fracture line [5]. The complications of TBF include facial nerve palsy (FN), hearing loss (HL), which may be conductive, sensorineural, or mixed, and have a cerebrospinal fluid (CSF) leak [6,7,8]. All these sequelae of TBFs may adversely affect quality of life (QOL) with a psychological, emotional and social impact.

To our knowledge, the number of studies referring to QOL in patients suffering from TBF are very restricted [9]. In this study, we report on patient-assessed outcomes following TBF in relation to audiovestibular symptoms and associated laboratory tests and to describe their impact on QOL.

## 2. Material and Methods

This study included 30 consecutive patients suffering from TBF and who were referred to our university tertiary center over a 2 year period (January 2017 to January 2019). Subjects were excluded if there was a known preexistent HL before TBF or other otological disease. Twenty-six males (87%) and four females (13%), aged 40y ± 17.85 and 40.5y ± 22.31, respectively, were retrospectively examined in our center. Their neurotological findings are shown in Table 1. The affected side was the left one in 15 patients (50%), the right one in 11 cases (36.7%), and bilateral in 4 patients (13.3%). Longitudinal TBF was present in 17 (56.6%) out of 30 patients, mixed TBF in 8 (26.6%) patients, and transverse TBF in 5 (16.6%) patients. The mechanisms of injury are presented as follows: motor vehicle accidents in 15 (50%) cases, severe fall (from height more than two meters) in 9 (30%) cases, a pedestrian accident in 3 (10%) cases, and falling from the patient’s height and assault in 3 (10%) cases.

A complete clinical history was obtained for all the patients, and the presence of tinnitus and vestibular symptoms were also noted. Otoscopy was also effectuated to detect any ear bleeding or CSF leak. High-resolution computing tomography of the temporal bone with multiplanar reconstructions was utilized to locate and categorize the fracture according to the fracture line trajectory. Pure-tone audiogram thresholds were obtained and hearing was classified as normal when the hearing threshold was at 0–20 dB, mild at 21–40 dB, moderate at 41–70 dB, severe at 71–90 dB, and cophosis at 91 dB or more. HL was also diagnosed as conductive, sensorineural, or mixed. 

The onset of traumatic facial palsy (FP) may be immediate within the hours following delayed injury due to secondary ischemia caused by edema within the bony canal. Moreover, the FP was evaluated based on its severity by the House Brackmann (HB) grading scale [10]. HB grade I was defined as a normal function, HB II as mild FP, HB III-IV as moderate FP, and HB V-VI as severe FP. The clinical signs of HB were for: Grade I, normal symmetrical function; grade II: Slight weakness noticeable only on close inspection, complete eye closure with minimal effort, and slight asymmetry of smile with maximal effort. Grade III: Obvious weakness but not disfiguring, not able to lift eyebrow, complete eye closure and asymmetric mouth movement with maximal effort. Grade IV: Obvious disfiguring weakness, inability to lift brow, incomplete eye closure and asymmetry of mouth with maximal effort. Grade V: Motion barely perceptible, incomplete eye closure and slight movement of corner of mouth. Grade VI: No movement and loss of tone.

All the patients initially underwent the electrophysiological tests, including the excitability test and electroneurography (ENoG), in order to evaluate the facial nerve function. During ENoG, the amplitude of the compound action potential was recorded via a surface stimulator (Amplaid MK12) with maximum current intensity, avoiding patient discomfort; a total nerve degeneration of facial nerve (>95%) within the first 2 weeks was considered “severe”, otherwise an ENoG result <95% was characterized as “non-severe”. When performing the excitability test (Myoton 2 Facial Nerve Stimulator), the current intensity via the probe was increased from zero to a threshold level sufficient to generate a regional muscle contraction. A difference of excitability of 3.5 mA or less between the normal and the affected side was defined as “normal”; a difference of excitability of 3.5 mA or greater between the two sides was defined as “diminished”; and “no response” characterized those from whom no facial reaction was obtained no matter how much current was applied. 

Patients suffering from traumatic sensorineural HL and/or tinnitus, facial palsy and dizziness following TBF were treated conservatively with intravenous dexamethasone (Decadron 8 mg × 3/day with schema for 10 days). Patients with conductive and mixed HL were further assessed with serial pure-tone audiograms. The CSF leak was treated with conservative measures (bed rest for 3 days with thromboprophylaxis, head elevation at 30 degrees, prophylactic antibiotics and lumbar drainage placement after the third day). No leak persisted for more than 7 days. 

Our patients participated from injury onset to the final follow-up over an 18-month period when no further clinical improvement could be expected for cranial nerves, such as for the facial function (even after facial nerve repair). At that time, the HB grading was reevaluated and a pure-tone audiogram was repeated. The assessment of sequelae and QOL was determined with the use of different validated questionnaires, including the Facial Disability Index [11], the Hearing Handicap Inventory [12], the Tinnitus Handicap Inventory [13] and the Dizziness Handicap Inventory [14]. 

The Facial Disability Index (FDI) is a 10-question instrument with two subcategories: five questions concerning the physical performance and five questions regarding social and well-being outcomes. Each category was counted using a six-point scale. Both of them were reduced to a 100-point score system, with 100 revealing intact function, whereas 0 corresponds to complete facial palsy [11].

The hearing handicap inventory for adults (HHI-A) is comprised of 25 items subdivided in two subcategories: the social and emotional one. There are three potential answers: “yes”, “sometimes”, and “no”. They are equal to 4, 2, and 0, respectively. Higher values indicate a more devastating perception of the hearing handicap. Scores ranging from 0–16 correspond to no handicap, 18–43 to a mild and moderate one, whereas 44 or more constitutes a significant handicap [12]. 

The Tinnitus Handicap Inventory (THI) is a 25-question survey. It quantifies the impact on functional, emotional and catastrophic subscales. The possible points are “yes”, “sometimes”, and “no”. They respond to 4, 2, and 0, respectively. Higher scores are associated with a more negative perception of tinnitus [13]. A THI score of 0 to 16 is classified as no or a slight handicap, 18 to 36 as mild, 38 to 56 as a moderate handicap, 58 to 76 as severe, and finally, 78 or more as a catastrophic handicap.

The main scope of the Dizziness Handicap Inventory (DHI) was the assessment of vestibular disability. It is a 25-item instrument which rates sequels of dizziness in the physical, functional, and emotional areas. There are three possible responses, including “yes”, “sometimes”, and “no”. The score each response receives is 4, 2, and 0, respectively. Higher scores indicate worse negative effects imposed by dizziness [14]. Scores ranging from 0–14 signified no handicap; 15–34, mild handicap; 36–52, moderate handicap; and more than 54, severe handicap.

### Statistical Analysis

To define the normality of the variables, we employed the Kolmogorov–Smirnov test. A comparison between numerical and ordinal variables was performed using the independent t-test when there were two of them, and a one-way ANOVA test when there were three or more. For post-hoc analysis correction according to Dunn-Bonferroni test was implemented. We extracted Spearman’s rank correlation coefficient in cases of nonparametric correlation. Linear regression was assessed using the predictors that showed a statistically significant correlation with the corresponding outcome variables. P-values less than 0.05 in two-tailed tests were considered statistically significant. SPSS v.25.0 software was used for our analysis.

## 3. Results

On initial presentation, 12 (40%) patients suffered from FP, 26 (86.6%) patients from HL, 21 (70%) from tinnitus, and 17 (56.6%) from dizziness (Table 1). Regarding the patients with FP, in five cases the TBF was longitudinal, in five it was mixed, and in two it was transverse. Two out of six patients with immediate FP underwent surgical decompression of the facial nerve via the transmastoid approach; one patient had postoperative HB III, and the other had HB IV. From the other four patients who were treated conservatively, three (75%) patients had mild FP, and the other patient had HB III.

The HL was conductive in 11 patients (36.6%), sensorineural in 8 (26.6%), and mixed in 7 (23.3%) patients. In longitudinal TBF, conductive HL occurred in nine patients, sensorineural HL in three, mixed HL in three, and there was no HL in two; regarding transverse TBF, conductive HL was noted in one patient, sensorineural HL in two, and mixed in two; regarding mixed TBF, conductive HL was found in one patient, sensorineural HL in three, mixed HL in two, and there was no HL in two. 

Dizziness was present in nine (52%) patients with longitudinal TBF, in two (40%) patients with transverse TBF, and in six (75%) patients with mixed TBF. Three patients, two with longitudinal TBF and one with mixed TBF, were affected by unilateral benign paroxysmal positional vertigo (BPPV). 

### 3.1. Facial Nerve Sequels

Eighteen months after the injury, three patients had moderate FP and six patients had mild FP (Table 1). Three out of the 12 patients who initially had moderate to severe FP after TBF had completely recovered. No patient had severe FP. Three out of six patients with immediate FP had mild FP, and the other three patients had moderate FP (two of them had been surgically treated); three out of six patients with delayed FP had mild FP, and the other three recovered completely. One patient, who initially had HB V, had synkinesia with HB III after conservative treatment.

At the long-term follow-up, the FDI-physical function score was significantly worse in patients who initially had severe and immediate FP, and/or no or diminished response in the excitability test (Table 2); at the follow-up, moderate or even mild FP was also significantly associated with a worse FDI-physical function score (Table 2). Regarding long-term FDI-social function (Table 2), patients with mixed TBF, who presented with immediate onset of FP and/or did not exhibit a response in the excitability test showed a worse score; at the follow-up, patients with moderate FP also demonstrated a reduced FDI-social function score. 

Male patients, who constituted the majority (87%) of our sample, experienced more FDI-physical and -social dysfunction compared to females. Age and the affected side (unilateral or bilateral TBF) did not have a statistical effect on both the FDI-physical and -social function.

### 3.2. Audiovestibular Sequels

From the 11 patients with conductive HL, six recovered to normal hearing, four to mild HL, and one to moderate HL. From the eight patients with sensorineural HL, one recovered to normal hearing, three to mild HL, two to moderate HL, one to severe HL, and one to profound HL. From the seven patients with mixed HL, two recovered to mild HL, three to moderate HL, one to severe HL, and one to profound HL.

Of the 30 participants with TBF who reported HL, the mean total HHI score was 22.9 (Table 3); accordingly, the mean total THI and DHI was 18.1 and 15.3, respectively. Patients with mixed HL had a worse HHI score compared to those with conductive HL, and patients with profound HL also had a reduced HHI score (Table 4). At the follow-up, the THI and DHI scores were higher in patients with moderate FP compared to patients with complete recovery of facial function (Table 4). Mixed fractures appeared to significantly worsen the HHI, THI, and DHI scores compared to the longitudinal TBF (Table 4). 

The regression analysis (Table 5) showed that the final HB (*p* < 0.001), onset of FP (*p* < 0.01) and age (*p* < 0.05) were negatively correlated with FDI-physical scores; the final HB was also negatively correlated with FDI-social function (*p* < 0.001). Moreover, the statistical analysis showed a positive correlation of FDI-physical function scores with the excitability test and the electroneurography’s results. The degree of hearing loss (final audiogram) was positively correlated with the HHI (*p* < 0.001), THI (*p* < 0.01) and DHI (*p* < 0.05) score. Finally, the type of fracture could statistically influence the HHI, THI, and DHI scores (*p* < 0.05). 

## 4. Discussion

It has been reported that the FP has an important impact on functional abilities and social interaction [15,16]; this was confirmed by our results, as long-term physical and social FDI scores were worse in more pronounced FP palsy (HB grade) (Table 2 and Table 5); however, no patient had severe FP in the long-term. Montava et al. [9], in 12 (27.9%) out of 43 patients suffering at onset from FP after TBF, did not find a significant correlation between the QOL and initial FP or long-term FP.

Nevertheless, the FP may contribute to psychological distress, which could result in social disability more easily than the functional impairment itself [17]; at follow-up, our patients with moderate FP had a poorer mean social FDI score than that of physical FDI (50.7 vs. 78.3, Table 2). As the face plays a major role during interpersonal communication, changes in the ability to smile and express emotions may predispose the patient to social isolation [18]. Physically impaired patients with FP displayed more inhibition in social behavior, and tended to withdraw from their peers [19]. Therefore, although the functional problems of FP might have been resolved, it is important that psychosocial factors are also addressed to maximize the patient’s satisfaction and their QOL [15,17]. Volk et al. [20] initially assessed (without follow-up assessments) the predictors for baseline FDI scoring in 116 (45%) out of 256 patients with Bell’s palsy and 93 (36%) patients with traumatic FP (interval onset of FP to assessment <90 days in 31% of cases, >90 days in 69% of cases); they supported the fact that older patients had a worse physical FDI score, females also had worse social FDI function, and a higher initial HB grade was independently correlated to a poorer physical FDI score. The same conclusion was reported in the study of Bylund et al. [21], who applied FDI measures in 96 patients suffering from Bell’s palsy, and found that women experienced more FP-related psychosocial dysfunction. However, in our study, although age was negatively correlated with the physical FDI score, male patients exhibited worse FDI scores compared to women, possibly due to their larger majority in our sample (87% vs. 13%).

Mixed TBF significantly influenced the social FDI compared to longitudinal fractures; this could be explained, in our series, by the increased proportion of initial moderate–severe HB in mixed TBF (62%) compared to longitudinal TBF (29%), which might lead the patients to long-term social isolation and psychological disabilities. As mixed TBF constituted more complex and severe fracture patterns containing both longitudinal and transverse components, it may injure the facial canal more frequently [22]. Ishman and Friedland [5], in a sample of 155 TBF, found an increase rate of FP in mixed fractures (25%) compared to longitudinal fractures (4%) and transverse fractures (13.9%). 

In our study, immediate FP carried a poor prognosis for satisfactory physical and social FDI (Table 2 and Table 5). The time of onset of FP after injury is critical for further management. Immediate FP, which is associated with an interruption of nervous conduction in a nerve transection or nerve compression by bone spicula may warrant surgical intervention [4,6,23]. However, in a study [23] comprising 174 patients with immediate FP after TBF, postoperatively, 28 (16%) had final HB I, 123 (71%) had HB II-V, and 11 (6%) HB VI, supporting the fact that surgical intervention did not appear to improve on the natural course of immediate FP; we agree with this, as 75% of our patients with immediate FP consequently had mild FP after conservative treatment and the two surgically treated patients had final HB III and IV. Facial function recovery among patients with delayed FP is almost always to HB I or II, precluding surgical decompression of the facial nerve [6,7,24].

The electrodiagnostic tests, including the ENoG and excitability test, have been widely used to predict facial nerve recovery after traumatic FP [3,6,7,24]. In post-traumatic FP, neural degeneration of the facial nerve correlates with poor recovery by more than 95%, especially when it occurs within the first 14 days after injury [25]. In our study, a severe degeneration in the initial ENoG had a significant impact on long-term physical FDI, reflecting functional disability due to neural conduction damage (Table 5). It has been advocated that patients presenting with severe and immediate FP, associated with more than 95% degeneration within 15 days after injury, may require surgical intervention [25]. Similarly, in our study, the excitability test was shown to statistically correlate with the outcome of facial function (Table 2 and Table 5). The excitability test also remains a valuable prognostication tool in FP, detecting the conductivity and the functional state of the facial nerve [26]. In a study [27] including 100 patients suffering from non-penetrating traumatic FP, 95% of patients achieved complete recovery with a normal excitability test; in the event of no response in the same test, 80% of patients were also reported to have poor recovery. In the case of severe traumatic lesion of the facial nerve, the nerve fibers cannot propagate electrically generated evoked potentials distal to the injury, even with minimal (excitability test) or maximal (ENoG) applied electrical current [27]. 

In our study, the proportion of patients complaining of HL, tinnitus and dizziness was relatively low regarding scores in the significant–severe range (26.7% for HHI, 13.3% for THI, 6.7% for DHI) (Table 3), showing that the impact of the above symptoms was not so pronounced on patients’ emotional and physical health. Regarding HL, almost 27% of cases returned to normal thresholds, especially in those with conductive HL; conductive losses from tympanic membrane perforation, hemotympanum, or suspected ossicular chain injury spontaneously improved with time to within acceptable limits. Higher HHI and THI values were significantly associated with profound HL at onset and follow-up in one or both ears (Table 4 and Table 5), which corresponded with poor communication, depression, social withdrawal, and isolation, resembling the negative impact of severe HL on the quality of life of elderly individuals [28]; even the unilateral profound HL may have a negative impact on HHI, with loss of binaural perception and substantial difficulties with listening in everyday situations, especially at social events [8,29]. Patients who have mild to severe sensorineural HL are usually treated with standard hearing aid amplification. For unilateral profound HL, contralateral routing of signal (CROS) hearing aids, bone conduction devices, and cochlear implants can restore hearing to deaf ears, thereby providing the patient access to binaural hearing, along with reduction of tinnitus [30]. Because head trauma often involves high-speed motor vehicle collisions, mixed fractures are frequently encountered [31]; as both longitudinal and transverse components implicate conductive and sensorineural HL, respectively [7], we expect that mixed fractures may affect both the HHI and THI indexes (Table 4 and Table 5).

The incidence rates of HL following TBF have been reported to be 26% to 57% for conductive HL, 14% to 23% for sensorineural HL, and 20% to 55% for mixed HL (3); our incidence of post-traumatic HL falls within or is quite closed to the published rates. According to our results, the HHI score was significantly worse in mixed than in conductive or sensorineural HL (Table 4). Similar findings have been shown in other studies [32,33], in which post-traumatic audiometric data were worse in mixed HL compared to conductive HL, mainly in the initial presentation but also at follow-ups. The mixed HL, combining the conductive with sensorineural malfunctions, may have a more negative impact on hearing. The perception of speech in noise is more complex in patients suffering from mixed HL, as the consequences of damage to the outer hair cells result in a loss in auditory resolution and speech clarity, especially in noise [34]; the use of bone conduction hearing implants has planned to overcome this hearing limitation [34]. 

It is assumed that cochlear damage may lead to neuroplastic reorganization of central auditory and nonauditory pathways [35]; these changes may initiate increased spontaneous firing rates and/or an increase in neural synchrony, resulting in tinnitus perception [36]. A stressful traumatic event, such as the TBF, can also trigger the development of posttraumatic stress disorder and tinnitus [37]. In our study, 70% of our patients affected by TBF suffered from tinnitus (Table 1), that might seem to be a symptom of lower-ranking importance; however, after recovering from other trauma sequela, it may persist as a condition influencing one’s quality of life [38].

At follow-up, the sensation of dizziness and unsteadiness was reported in 56.6% of cases (Table 1). The DHI score was significantly higher in patients affected by mixed TBF (Table 4) and was relatively better compared to the other handicap inventory mean scores (Table 3). In most of these patients, the symptoms were aggravated by sudden, rotatory head movements and some described balance problems during walking. Singh et al. [32], in his study of 50 patients of head injury, reported disturbed balance and vertigo in 29 patients (58%). Initially, three of our patients presented with post-traumatic otolithic detachment (BPPV), which was cured after liberatory maneuvers. In a study [6] which enrolled 141 cases with TBF, vertigo occurred in 86 (67.18%) patients, of which 64 patients had BPPV in the form of canalolithiasis and 22 had an acute vestibular deficit, treated with pharmacotherapy and vestibular rehabilitation. Usually, vestibular symptoms such as dizziness and unsteadiness are self-limiting and resolve within 3–12 months [6,7] due to the central vestibular compensation effect, which occurs following brainstem injury (because of vestibular nuclei damage).

## 5. Conclusions

In the long-term, traumatic FP has an important impact on functional abilities and social interaction. Therefore, although the functional problems of FP might be resolved, it is important that psychosocial factors are also addressed to maximize patients’ level of satisfaction and their QOL. From prognostic factors, mixed TBF, as more complex and severe fracture patterns, significantly influenced the social FDI, HHI, THI and DHI scores compared to longitudinal fractures. Following TBF, the mixed HL, combining the conductive with neural malfunctions, can have a negative impact on hearing. The post-traumatic profound HL in one or both ears may also cause worse HHI, THI, and DHI values, which corresponds with poor communication, depression, social withdrawal, and isolation. Immediate FP carried a poor prognosis for long-term satisfactory physical and social FDI, which can be initially evaluated in a better way by the ENoG and excitability tests.

## Figures and Tables

**Table 1:** Neurotological findings in patients (*n* = 30) initially and at follow-up after temporal bone fracture. Into brackets: number of patients. HL: hearing loss, ENoG: electroneurography of facial *n*.

Traumatic facial Palsy	Immediate (6)	Delayed (6)			
Type of fracture	Longitudinal (17)	Transverse (5)	Mixed (8)		
Initial HB grade	I (18)	II (0)	III-IV (8)	V-VI (4)	
Final HB grade	I (21)	II (6)	III-IV (3)	V-VI (0)	
ENoG result	non-severe (27)	severe (3)			
Excitability test	Normal (19)	Diminished (5)	No response (6)		
Type of hearing loss	Conductive (11)	Sensorineural (8)	Mixed (7)		
Initial pure-tone audiogram	Normal (7)	Mild HL (7)	Moderate HL (10)	Severe HL (3)	Profound HL (3)
Final pure-tone audiogram	Normal (9)	Mild HL (9)	Moderate HL (6)	Severe HL (3)	Profound HL (3)
Tinnitus (initial)	Yes (21)	No (9)			
Dizziness (initial)	Yes (17)	No (13)			
Ear bleeding	Yes (24)	No (6)			
CSF leak	Yes (3)	No (27)			

**Table 2:** Mean values and standard deviation of neurotological parameters for FDI-physical score and FDI-social score (* *p* < 0.05, ** *p* < 0.01, *** *p* < 0.001).

		Facial Disability Index-Physical Function	Significance	Facial Disability Index-Social Function	Significance
Gender	male	95.5 ± 8.7	*	87.4 ± 23.7	*
	female	100 ± 0		100 ± 0	
Age	young	99.3 ± 1.9		91.4 ± 22.7	
	adult	95.1 ± 9.5		88.7 ± 21.9	
	older	95 ± 8.7		87.2 ± 28.6	
Afflicted side	left	96 ± 8.1		89.3 ± 22.4	
	right	97.5 ± 5.5		89.5 ± 23.5	
	bilaterally	92.5 ± 15		87 ± 26	
Initial HB (I–VI)	complete recovery	99.7 ± 1.2	***	96.4 ± 15.1	
	mild facial palsy	-	complete-severe ***	-	
	moderate facial palsy	95.9 ± 6.7	moderate-severe ***	79 ± 29.1	
	severe facial paralysis	80 ± 10.8		76 ± 27.9	
Final HB (I–VI)	complete recovery	99.8 ± 1.1	***	97 ± 14	**
	mild facial palsy	92 ± 8.8	complete-mild **	80.7 ± 30	complete-moderate **
	moderate facial palsy	78.3 ± 10.4	complete-moderate ***	50.7 ± 4.6	
	severe facial paralysis	-	mild-moderate **	-	
Excitability test	normal	99.7 ± 1.1	**	96.6 ± 14.7	**
	diminished	89 ± 13.4	normal-diminished **	89.6 ± 23.3	normal-no response **
	no response	90.3 ± 10.1	normal-no response *	64.7 ± 27.9	
Initial ENoG of facial nerve	non severe	97.3 ± 6.8	*	91.7 ± 20.5	
	severe	85 ± 13.2		65.3 ± 30	
Type of fracture	transverse	100 ± 0		88 ± 26.8	**
	longitudinal	96.9 ± 7.3		97.6 ± 10.4	longitudinal-mixed **
	mixed	91 ± 11.4		68 ± 30.2	
Onset of facial palsy	no	99.7 ± 1.2	***	96.2 ± 15.5	*
	delayed	99.3 ± 1.9	no-immediate ***	91.4 ± 22.7	no-immediate *
	immediate	82 ± 9.3	delayed-immediate ***	66 ± 26.6	

**Table 3:** Number of patients (Nb, %), mean and median scores (SD, standard deviation) for each of the Hearing Handicap Inventory (HHI), Tinnitus Handicap Inventory (THI) and Dizziness Handicap Inventory (DHI) questionnaires at long-term follow-up after temporal bone fracture.

	Nb of Patients	%
HHI		
No handicap	16	53.3
Mild to moderate handicap	6	20.0
Significant handicap	8	26.7
Total	30	100.0
Mean ± SD		22.9 ± 26.5
Median [25%, 75%]		13 [0, 72]
THI		
No or slight handicap	19	63.4
Mild handicap	6	20.0
Moderate handicap	1	3.3
Severe handicap	3	10.0
Catastrophic handicap	1	3.3
Total		100.0
Mean ± SD		18.1 ± 24.7
Median [25%, 75%]		7 [0, 72]
DHI		
No handicap	19	63.3
Mild handicap	6	20.0
Moderate handicap	3	10.0
Severe handicap	2	6.7
Total	30	100.0
Mean ± SD		15.3 ± 21.0
Median [25%, 75%]		6 [0, 62]

**Table 4:** Mean values and standard deviation of neurotological parameters for Hearing Handicap Inventory (HHI), Tinnitus Handicap Inventory (THI) and Dizziness Handicap Inventory (DHI). HB: House Brackmann grading, CSF: cerebrospinal fluid (* *p* < 0.05, ** *p* < 0.01).

		HHI	Significance	THI	Significance	DHI	Significance
Type of fracture	transverse	32 ± 24.8	*	21.2 ± 19.1	*	12.4 ± 12.2	**
	longitudinal	12.4 ± 16.8	longitudinal-mixed *	9.7 ± 18.4	longitudinal-mixed *	6.9 ± 13.3	transverse-mixed *
	mixed	43.1 ± 36		37.4 ± 32.9		39.1 ± 25.4	longitudinal-mixed **
Initial HB (I–VI)	complete recovery			11.1 ± 18.1		11.4 ± 14.3	
	mild facial palsy			-		-	
	moderate facial palsy			27.5 ± 26.8		21 ± 25.6	
	severe facial paralysis			30.5 ± 40.1		21.5 ± 36.6	
Final HB (I–VI)	complete recovery			11.2 ± 18	*	11.4 ± 14.5	*
	mild facial palsy			27 ± 25.3	complete-moderate *	13.7 ± 20.4	complete-moderate *
	moderate facial palsy			48 ± 43.9		46 ± 40.4	
	severe facial paralysis			-		-	
Initial Audiogram dB	normal hearing	97.4 ± 6.8	*	2.9 ± 7.6		13.1 ± 14.9	
	mild HL	95.7 ± 9.3	normal-profound HL*	22 ± 31.2		16 ± 22.9	
	moderate HL	94 ± 10.5		17.2 ± 26.7		11 ± 23.5	
	severe HL	100 ± 0		18 ± 15.6		10.7 ± 15.1	
	profound HL	96.7 ± 5.8		47.3 ± 11		38 ± 22.3	
Final Audiogram dB	normal hearing	98 ± 6	*	3.6 ± 7.3		11.1 ± 13.7	
	mild HL	96.7 ± 8.3	normal-profound HL *	18.7 ± 27.8		12.4 ± 21	
	moderate HL	95 ± 7.7		16 ± 17.3		10 ± 13.5	
	severe HL	90 ± 17.3		37.3 ± 44.1		36 ± 36.7	
	profound HL	96.7 ± 5.8		44.7 ± 15.3		26.7 ± 31.9	
Initial ear bleeding	yes	95.1 ± 9		19 ± 25		13.9 ± 21.6	
	no	100 ± 0		14.3 ± 25.2		21 ± 19	
Initial dizziness	yes	96.5 ± 8.6		18.5 ± 26.4		19.8 ± 23.2	
	no	95.5 ± 8		17.5 ± 23.3		9.5 ± 16.8	
Initial CSF leak	yes	90.7 ± 9		35.3 ± 20		27.3 ± 31.6	
	no	96.7 ± 8.1		16.1 ± 24.7		14 ± 19.9	
Initial tinnitus	yes	96.7 ± 7.8		19.2 ± 25.2		13.8 ± 21.7	
	no	94.7 ± 9.5		15.3 ± 24.7		18.9 ± 19.8	
Type of hearing loss	normal	95.5 ± 9	*	5 ± 10		16 ± 15.7	
	conductive	96.8 ± 7.5	conductive-mixed *	11.1 ± 22.6		8.2 ± 16.9	
	sensorineural	98.1 ± 3.7	sensorineural-mixed *	20.5 ± 25.7		20 ± 23.5	
	mixed	92.9 ± 12.5		33.7 ± 27.7		20.9 ± 26.9	

**Table 5:** Multiple regression results for outcome variables of facial disability index (FDI)-physical, FDI-social, hearing handicap inventory (HHI), tinnitus handicap inventory (THI), and dizziness handicap inventory (DHI). ^a^ No response-diminished-normal, ^b^ Severe-non severe, ^c^ No-delayed-immediate, ^d^ Normal-mild-moderate-severe-profound, ^e^ Transverse-longitudinal-mixed. HB: House Brackmann grading, ENoG: electroneurography, FL: facial nerve, dB: decibels, (* *p* < 0.05, ** *p* < 0.01, *** *p* < 0.001).

	B	95% CI for B		SE B
		LL	UL	
FDI-physical function				
Final HB	−14.996 ***	−20.629	−9.363	2.729
Excitability test ^a^	6.830 **	3.318	10.342	1.702
ENoG result ^b^	9.582 **	2.797	16.367	3.288
Onset of facial palsy ^c^	−4.556 **	−7.864	−1.247	1.603
Age	−0.079 *	−0.158	−0.001	0.038
FDI-social function				
Final HB	−0.079 ***	−31.163	−11.382	4.828
HHI				
Final Audiogram (dB) ^d^	13.300 ***	7.230	19.371	2.959
Type of fracture ^e^	13.492 *	1.131	25.853	6.024
THI				
Final Audiogram (dB) ^d^	11.039 **	5.107	16.971	2.891
Type of fracture ^e^	14.566 *	2.487	26.646	5.887
DHI				
Type of fracture ^e^	17.894 **	7.431	28.358	5.099
Final Audiogram (dB) ^d^	6.461 *	1.323	11.600	2.504

## Data Availability

The data presented in this study are available upon request from the corresponding author.
